# An exploratory study of illegal gamblers in Hong Kong

**DOI:** 10.1186/s40405-017-0030-7

**Published:** 2017-12-02

**Authors:** Andrew Tessler, Kareen El Beyrouty, Natasha Crapnell

**Affiliations:** 1 BIS Oxford Economics, Level 8, 99 Walker St., North Sydney, 2060 Australia; 2 Ricardo Energy and Environment, 30 Eastbourne Terrace, London, W2 6LA UK; 3 31 Cecil Road, Weston Super Mare, BS23 2NU UK

**Keywords:** Illegal gambling, Problem gambling, Excessive gambling, Hong Kong

## Abstract

This study investigates the nature and behaviour of illegal gamblers in Hong Kong. A face-to-face street survey of 512 gamblers was conducted in Hong Kong between September and December 2015 with supplementary convenience sampling allowing for analysis of a total sample of 103 illegal gamblers. 56% of illegal gamblers recorded results consistent with this study’s definition of ‘excessive gambling’ [i.e. moderate risk and problem gamblers under the Problem Gambling Severity Index (PGSI)]. 81% of surveyed illegal gamblers were male, 77% were aged between 30 and 49 and 67% were in blue collar occupations. Illegal gamblers bet more frequently on both legal and illegal games than their legal counterparts and spent more when they did bet. While this research did not indicate the direction of causality between illegal and excessive gambling, international work (de Bruin et al. in verslingerd aan meer dan een spel: Een onderzoek naar de aard en omvang van kansspelproblematiek in Nederland, WODC/CVO, Utrecht, http://www.lexandgaming.eu/nl/wp-content/uploads/2015/01/Verslingerd-aan-meer-dan-een-spel.pdf, 2005; Binde in What are the most harmful forms of gambling? Analysing problem gambling prevalence surveys, http://www.utbildning.gu.se/digitalAssets/1327/1327132_cefos-wp12.pdf, 2011) suggests that excessive gamblers are drawn to illegal gambling. Reform could allow excessive gambling by illegal gamblers to be better addressed and initial work suggests some financial benefits to Hong Kong.

**Electronic supplementary material:**

The online version of this article (10.1186/s40405-017-0030-7) contains supplementary material, which is available to authorized users.

## Background

Gambling is a regulated market in Hong Kong. The Hong Kong Jockey Club (HKJC) is the only authorised gambling operator licensed by the Hong Kong government, offering gambling options such as horse race betting, football betting and a lottery. However, a substantial illegal market for gambling exists, estimated at HK$12 billion per annum (HKJC 2017). These funds flow to unlicensed operators in Hong Kong, offshore operators outside of Hong Kong (excluding Macau casinos) and online operators both inside and outside Hong Kong. There are also potential links with organised crime. Indeed Wang and Antonopoulos (2015) cite research by Varese indicating that 35% of criminal groups regard gambling as a key source of revenue. The HK$12 billion in estimated illegal gambling revenues can be compared to the HK$36 billion in legal gambling revenues reported by the HKJC in 2015–2016 (HKJC 2016). Taken together, these figures suggest a total (legal and illegal) Hong Kong gambling market of HK$48 billion, some 25% of which involves illegal transactions.

The illegal gambling market would therefore appear to be substantial. It also operates outside of any official constraints and, by definition, involves criminal behaviour. This raises the issue of who illegal gamblers are, what sort of impact their activities are having (on themselves and others) and what can be done to address them.

Because of its nature and the difficulty of eliciting responses, very few past studies have investigated illegal gambling. Nonetheless, these studies do shed some light on the nature of illegal gamblers, chiefly related to the issue of gambling addiction. A major study of Dutch gamblers by de Bruin et al. (2005) provided some clues on the behaviours and characteristics of illegal gamblers, finding that illegal gambling stands out from other categories of gambling in terms of its high proportion of excessive gamblers.^1^

They conclude that illegal gambling clearly appeals to gambling addicts (less so to recreational players) and that it offers a certain attraction to risky and problematic players. The authors also find that Dutch illegal gambling is largely a middle aged male phenomenon. Binde’s (2011) meta-analysis of 13 European countries and major English speaking ones finds that ‘unregulated’ (illegal) gambling had a strong relationship with excessive gambling and that illegal gambling attracted those with a great interest in gambling who were not satisfied with the range of legal options. Volberg’s (2001) work in Sweden finds that excessive gamblers were statistically more likely than non-excessive ones to wager on illegal games, while Volberg’s (1996) work in New York found that the forms of gambling with the highest rate of excessive gambling were illegal ones.

However, while it is useful to understand the international context, no detailed study specifically analysing the habits of illegal gamblers appears to have been undertaken in Hong Kong.^2^

Nonetheless, this past work does suggest that a substantial proportion of illegal gamblers may also be excessive gamblers. If indeed this is the case, then illegal gamblers may suffer from many of the problems that beset excessive gamblers.

These problems have been well documented both internationally and within Hong Kong. While undertaken some time ago, work by Australia’s Productivity Commission (1999) is notable for its scale and comprehensive nature with the Commission suggesting that approximately 80–85% of a variety of social issues typically associated with excessive gamblers (depression, anxiety, poor health, reduced job productivity, high job turnover, substance abuse, suicide or suicidal thoughts, family and relationship problems and financial problems) could be attributed to excessive gambling.^3^ This work also suggests that gambling-related problems have far reaching effects on families and relatives. The Commission’s National Gambling Survey found that nearly one in ten excessive gamblers reported a split up with partners due to gambling, while one quarter of excessive gamblers seeking counselling reported the dissolution of a relationship due to gambling (Productivity Commission 1999).

Retrospective work by Petry (2003) of 347 excessive gamblers in the United States who had attended gambling treatment centres found high rates of psychiatric and related problems within the study group. Work by Kunzi et al. (2004) on 335 excessive gamblers attending counselling and therapy services in Switzerland found that family and personal relations were ‘seriously impaired’ as a result of gambling addiction though causal linkages were found to be complex for a range of personality disorders.

de Bruin et al. (2005) also indicated that excessive gamblers in general were found to suffer more from psychological problems, depression and psychiatric problems than was the case for recreational gamblers. They were also more likely to drink, smoke and take drugs than recreational gamblers and commit crimes such as theft or fraud.

However links between excessive gambling and social problems may be complicated by bi-directional influences. An international meta-analysis of excessive gambling and intimate partner violence by Dowling et al. (2016) supports the idea that excessive gambling may precipitate intimate partner violence due to stress and financial problems but also that excessive gamblers could be victims of intimate partner violence themselves, with such violence precipitating excessive gambling.

In Hong Kong, Shek et al. (2012) found that three-quarters of pathological gamblers in Hong Kong treatment centres with mood disorders and two-fifths of those with lifetime adjustment disorders reported these problems after the onset of pathological gambling.^4^ Hong Kong work has also touched on the issue of illegal gambling. While not proving causality Wong et al. (2014) found a distinction between legal and illegal gamblers within the population of excessive gamblers, with illegal excessive gamblers found to be statistically more likely to have suicidal thoughts than legal excessive gamblers.

Chan et al. (2016) provide a comprehensive analysis of research into excessive gambling in Hong Kong, drawing on local and overseas sources. This work cites a series of studies in Hong Kong and Macau, examining the validity of the Blaszczynski and Nower Pathway Model for pathological gambling. The single largest group of excessive gamblers is found to be behaviourally conditioned gamblers—i.e. those who are free of psychopathology prior to the acquisition of gambling habits, though the work also identifies smaller groups (emotionally vulnerable gamblers and antisocial-impulsivist gamblers) who have underlying problems apart from gambling addiction.

Other Hong Kong work has explored the explicit links between excessive gambling, debt and suicide, with work by Wong et al. (2008) on suicide in the 30–49 age range finding that 14.1% of suicide cases were pathological gamblers and that the presence of unmanageable debt and unemployment stood out as the most significant predictors of suicide risk. Work by Yip et al. (2007) suggests that illegal gambling was reported as a cause of debt accumulation in 9% of debt-related suicide cases.

The above literature on both illegal gambling and issues associated with excessive gambling in general raises a number of issues. There is some international evidence that many illegal gamblers are also excessive gamblers and both local and international evidence points to the social problems affecting excessive gamblers (though causality remains a complex issue). However, we have little evidence about the demographics or betting characteristics of illegal gamblers in Hong Kong. We don’t know much about their motivations for gambling or the susceptibility of Hong Kong illegal gamblers to excessive gambling and its attendant social issues, though work by Wong et al. (2014) and Yip et al. (2007) offers some tentative clues. A corresponding set of issues relates whether gamblers themselves engage in other illegal activity. Finally, the phenomenon of illegal gambling raises the question of the social, financial and economic effects of liberalisation (or conversely the effects of not undertaking such regulatory reform).

This exploratory study is a first step rather than a last word and cannot seek to fully address all of these issues. Rather, it represents an initial attempt to scope some of these issues within Hong Kong. The lack of knowledge about illegal gamblers—and the illegal market in general—represents an obvious research gap, while the potential size of the market suggests this is a substantial issue. Understanding the nature of illegal gamblers, and the market in which they operate, is therefore an important research question.

More specifically, if, as some of the literature cited above suggests, many illegal gamblers are also excessive gamblers than this represents a substantial social issue. This is especially so given the problems associated with excessive gambling identified in the literature. Given this, past literature in the field allows for the development of several hypotheses about the potential nature of Hong Kong’s illegal gamblers:

### **H**_**1**_

Illegal gamblers are more likely to be avid gamblers, betting larger amounts and betting more frequently than their legal counterparts.

### **H**_**2**_

Illegal gamblers are more likely to be excessive gamblers than is the case for legal gamblers.

### **H**_**3**_

Illegal gamblers are more likely to suffer from social problems (e.g. depression, work difficulties, family problems, financial problems) than legal gamblers.

In order to test these hypotheses, this paper reports on the results of a survey of legal and illegal gamblers conducted in Hong Kong between September and December 2015. The aim of the survey was to understand the nature of illegal gamblers, in particular their characteristics, betting habits, susceptibility to excessive gambling and potential social and financial difficulties. Some of the survey results are then used to explore the ancillary issue of the potential effects of regulatory reform. The results of this work were originally reported in Oxford Economics (2016).

## Method

### Participants

Oxford Economics employed specialist survey providers Ipsos to undertake a ‘main survey’ of 512 gamblers (both legal and illegal) in Hong Kong between September and December 2015.^5^ A standardised survey questionnaire was developed for this purpose, using both self-administered and interviewer-administered questions.

A booster survey focussing on illegal gamblers yielded an additional 88 illegal gamblers allowing for the results of 497 legal and 103 illegal gamblers (600 in all) to be compared.^6^

Interviewees in the main survey were based on age quotas using past Hong Kong gambling survey data. Age bands were used for this purpose, with the youngest band (15–17) containing 1.2% (six interviewees) of the total and the oldest (60 or above) containing 23.6% (121). Some 19.2% (102) participants in the main survey lived on Hong Kong Island, with 30.5% (166) living in Kowloon and 49.6% (242) in the New Territories.

The booster survey used the same age bands as the main survey, with 4.9% of participants (5) aged 15–17 but none aged 60 or above. Some 25.2% (26) participants were aged 30–39 while 47.6% (49) were aged 40–49. As discussed below, illegal gambling would appear to be very much a middle aged phenomenon.

### Instrument

The main and booster surveys were undertaken using a face-to-face format, with forms in Traditional Chinese and interviewers administering them in Cantonese.

Surveys were conducted according to the ESOMAR code of conduct. Accordingly, interviews were conducted only among those of ages 15 or above and were only conducted among those who provided consent. Respondents were given the option to refuse or terminate the interview. Sensitive questions (including PGSI questions) were self-administered by respondents using tablets so information was not divulged to interviewers. All data collected was anonymised and no identifiers were delivered as part of the raw data. A copy of the survey questionnaire (Additional file 1) is available at the link below.

The survey consisted of three main sections:

1. A screening section with questions on gender, age, residential location and various forms of gambling participation inside and outside Hong Kong.
2. The main section of the survey which asked respondents a series of questions about gambling frequency and type, bet amounts, attractors to gambling, changes in the amount bet in the event of a variety of liberalisation options, excessive gambling behaviours (consistent with the PGSI scale) and if they had ever suffered social/financial problems due to gambling. The use of a PGSI scale was based on its properties as a valid and reliable instrument after consultation with Dr Irene Lai Kuen Wong of the Hong Kong Polytechnic University. The social and financial questions were developed based on a review of the questions developed by industry experts for the Productivity Commission’s (1999) *National Gambling Survey* and its findings on the key areas of social and financial cost.
3. A demographic section with questions on education, marital status, household size, employment status occupation and income.

### Procedure

Street intercept interviews were conducted in major commercial areas of the Special Administrative region (SAR), using a quota sampling strategy. Age quotas based on the most recent survey of Hong Kong gamblers then available (Hong Kong Polytechnic University 2012) were used in collecting the sample. In addition, respondents were recruited by reference to the SAR’s three main living districts (Hong Kong Island, Kowloon and the New Territories), using the most recently available Hong Kong population census on district population (Hong Kong Census and Statistics Department 2013).

Administration of the main survey yielded 15 illegal gamblers. The booster study was then conducted to increase the number of illegal gamblers using a convenience sampling approach in the vicinity of venues known to be frequented by illegal gamblers. The booster survey used the same survey questionnaire as the main survey, employing both self-administered and interviewer-administered questions.

Accordingly, the analysis below both assesses the characteristics of illegal gamblers indicated by the survey work and offers some comparisons between this sub-population and the sub-population of legal gamblers.^7^

### Data analysis

The survey research design was undertaken through cooperation between Oxford Economics, Ipsos and Dr Wong.

Survey execution and raw survey data collection were undertaken by Ipsos. Oxford Economics analysed the data with a view to identifying any key characteristics of illegal gamblers. A series of Chi Square tests were run by Oxford Economics in order to identify differences between legal and illegal gamblers in key demographic and social categories and in order to help answer the three hypotheses advanced above.^8^ We also interpreted our results in the context of an extensive literature review.

## Results

### Excessive gambling

The main and booster surveys examined the characteristics of both legal and illegal gamblers, as well as ‘excessive gamblers’ as defined with reference to the PGSI. The nature of the convenience sample approach adopted in the illegal booster survey naturally calls for caution in inferring that the results for illegal gamblers are representative of *all* illegal gamblers in Hong Kong.^9^ This caveat is also true for the other sections discussing illegal gambler characteristics below.

Nonetheless, this approach is consistent with de Bruin et al. (2005) who undertook a large scale survey in the Netherlands. Their work used a range of methods including recruitment at gambling venues to interview gambling sub-populations, given sufficient numbers could not be recruited through random sampling methods.^10^

The survey found that illegal gamblers were more likely to be excessive gamblers than was the case for legal gamblers. In particular, out of the population of illegal gamblers (n = 103), 56% of illegal gamblers could be defined as excessive gamblers compared to 15% of legal gamblers, (n = 497) with the difference between the two being statistically significant (Table 1).^11^

**Table 1:** Excessive gambling: legal versus illegal gamblers

Category	Legal gamblers: % (n = 497)	Illegal gamblers: % (n = 103)
Non-excessive gamblers	62	28
Low level excessive gamblers	24	17
Excessive gamblers	15	56

### Gender and age

The survey results indicate that 81% of illegal gamblers were male. 21% of illegal gamblers began gambling before the age of 18, compared with 11% of legal gamblers, a statistically significant difference.

Illegal gambling was found to mainly be a middle aged phenomenon. 77% of illegal gamblers were aged between 30 and 49 (compared to 33% of legal gamblers) while 18% were aged between 50 and 60. Only 5% were aged 18–29. No illegal gamblers were recorded over the age of 60.

### Marital status

There was little difference in marital status between illegal and legal gamblers, with 67% of illegal gamblers being married, compared to 71% of legal ones. However, one might expect relatively high rates of marriage, given that many illegal gamblers are middle aged.

### Employment

While 14% of both legal and illegal gamblers were classified as white collar workers, 62% of illegal gamblers were in blue collar occupations, as compared to 38% of legal ones. Interestingly, 89% of illegal gamblers reported having jobs. However, given the age structure of illegal gamblers (dominated by middle aged men, no survey respondents over 60) this is not especially surprising.

### Demographic summary

Taken together, the above survey results suggest that a typical illegal gambler in the SAR is a middle aged male in a blue collar job. It is also interesting to note that illegal gambling appears to peak in middle age, consistent with the findings of de Bruin et al. (2005).To the extent that illegal gambling involves social harms many of these harms would therefore be visited upon middle aged men and their families.

### The nature of the illegal market

The survey also examined which betting products illegal (and legal) gamblers placed bets on and how much they bet over the past year. Illegal gamblers were particularly drawn to illegal football betting (84% of illegal gamblers placed illegal football bets in the past year) with illegal horse racing close behind (80%). Fewer illegal gamblers (25%) had placed bets at illegal casinos in the past year, perhaps because of the range of legal options available at Macau.

Whether they bet illegally or not, illegal gamblers also bet more frequently than legal ones. 55% of illegal gamblers, gambled at least 3–4 times a week as opposed to 20% of legal gamblers (Table 2). The difference in gambling frequency between legal and illegal gamblers was found to be statistically significant.

**Table 2:** Frequency of gambling activity: legal versus illegal gamblers

Category	Legal gamblers: % (n = 497)	Illegal gamblers: % (n = 103)
Every day	2	9
5–6 times a week	5	19
3–4 times a week	13	27
Less than 3–4 times a week	80	45

Illegal gamblers also appear to spend far more than legal gamblers. When asked about their gambling spend over the past month (regardless of whether the bets were legal or illegal), illegal gamblers indicated that they spent most on casinos (mean = HK$8400, compared to HK$4100 for legal gamblers), sports betting (HK$5400 compared to HK$1200) and horse racing (HK$5400 compared to HK$1700). Spend differences between legal and illegal gamblers were statistically significant in all three cases.^12^

These facts are also consistent with international evidence that illegal gamblers tend to be particularly avid gamblers.

### Motivations for illegal gambling

Both illegal and legal gamblers were asked to select the three most important attributes they looked for when placing bets. By far the most important attribute to illegal gamblers was the fact that illegal gambling provided attractive odds (69% cited this against 34% of legal gamblers), followed by the ease of placing bets (46 vs. 40%) and the ability to bet any time (42 vs. 25%) This is again consistent with the profile of illegal gamblers as being particularly avid gamblers, consistent with the past studies such as Binde (2011) and de Bruin et al. (2005).

### Social impact of illegal gambling

To the extent that excessive gamblers are overrepresented within the ranks of illegal gamblers, one might expect that many of the problems faced by excessive gamblers might affect a large proportion of illegal gamblers.

While there is no simple way around the question of causality, highlighted earlier, the survey sought to deal with this by asking respondents whether they faced any personal or financial problems *due to their gambling* in the last 12 months.

The results are summarised in Table 3.

**Table 3:** Events experienced due to gambling in the last 12 months

Category	Legal gamblers: (n = 497) %	Illegal gamblers: (n = 103) %
Depression	2	1
Loss of time from work or study	1	4
Breakdown of relationship or divorce	1	5
Going into debt	1	4
Emotional distress of partners or children	1	6
Borrowing from unofficial money lenders	0	1
Loss of employment	0	1
Obtaining money illegally in order to gamble	0	2
One or more of the above	4	14

The number of respondents indicating that they suffered from any one difficulty is relatively small. However, while the great majority of all types of gamblers (legal and illegal) reported that they *did not* experience any of the problems they were asked about, the *overall proportion* of legal gamblers reporting that they *did* experience any of these problems due to their gambling (4%) is lower than that for illegal gamblers (14%). The difference between the two groups is statistically significant.^13^ Despite the limited sample size of illegal gamblers, the relatively high proportion reporting at least one negative social or financial issue is notable.

These results relate to all gamblers rather than excessive ones. It is possible to speculate that the illegal gambling results are driven by the high proportion of excessive gamblers who are also illegal gamblers, though further work would be necessary to establish this. In the interim, the results provide some tentative suggestion that illegal gamblers in general may be more susceptible to a range of personal and financial issues than their legal counterparts.

These issues, in turn, lead to considerations of how illegal gamblers finance their habit. In common with many other jurisdictions, Hong Kong bans offering credit in its legal gambling institutions, though of course no such rules apply to the illegal sector. In order to gauge the relative attractiveness of credit, the survey suggested a hypothetical scenario of legally offering credit in gambling. Some 19% of illegal gamblers indicated they would increase their monthly gambling spend in response to such a policy, compared to only 2% of legal gamblers. In addition, illegal gamblers indicated that they would increase their spend by HK$3005 per month in response to such a development, compared to HK$1150 for legal gamblers—i.e. by nearly three times the amount cited by legal gamblers.

The higher spending of illegal gamblers in general and their greater interest in credit raises the issue of their susceptibility to loan sharks and/or criminal activity in order to finance their habits. As indicated in Table 3, very few illegal gamblers indicated that they had borrowed from unofficial money lenders or obtained money to gamble illegally. Given the small numbers involved, no definitive evidence can be drawn from the survey. However, Wong et al. (2010) found that loan sharks were an important source of finance for pathological gamblers in Hong Kong and that threats from them may have been associated with suicides.

Likewise, while the illegal gamblers surveyed did not admit to committing crimes to finance their habit, Lahn and Grabosky (2003) found that one in four Australian pathological gamblers and one in ten problem gamblers had undertaken gambling-related illegal activity at some point. This work still leaves open the possibility that those committing crimes were more likely to gamble. However, after controlling for such causality, Clark and Walker (2009) found that excessive gamblers have a 17% higher likelihood of committing a serious crime.

Given the overrepresentation of excessive gamblers amongst illegal gamblers, these figures are sobering. Albanese (2008) makes the point that the social stigma associated with illegal gambling makes resulting financial pressures more ‘non-sharable’ than those occurring through legal gambling. His work implies that excessive gamblers who run up debts with illegal operators may be particularly likely to engage in crimes such as fraud.

A separate issue concerns the fact that illegal gambling is controlled by criminal syndicates. The survey underlying this paper concerned illegal gambling patrons rather than operators. However, in understanding the range of impacts of illegal gambling it is worth asking what ‘the house’ does with the proceeds. Wang and Antonopoulos (2015) suggest that 35% of criminal groups regard gambling as a key source of revenue. As illegal bookmakers do not pay fees or taxes, the activity can be highly profitable. Further, to the extent that funds are laundered and sent offshore, they do not contribute to the financial well-being of Hong Kong.

The fact that illegal operators, by definition, operate outside the conventional justice system also compels them towards violence, since the legal system cannot be used for collection of unpaid debts and indeed the collection of *any* gambling debt is illegal in mainland China (Wang and Antonopoulos 2015).

### The impact of reducing illegal gambling in Hong Kong

The survey results and international literature discussed above raises the issue of reform. Three aspects are relevant:

- The social implications of liberalising current gambling arrangements.
- The financial implications of gambling liberalisation.
- The economic implications of gambling liberalisation.

The evidence that a large proportion of illegal gamblers also appear to be excessive gamblers is noted above.

Excessive gambling may, of course, occur among both legal and illegal gamblers. However, the legal world offers an array of ‘brakes’, which seek to guard against the cultivation of damaging behaviours which could lead to or exacerbate excessive gambling. For example, those gambling through the HKJC are exposed to initiatives such as:

- Physical, online and TV programs aimed at promoting responsible gambling.
- A ban on credit in gambling.
- Exclusion or self-exclusion.
- Online self-assessment tools and finance plans to manage gambling.
- Information about excessive gambling counselling and treatment centres.
- Controls on underage gambling.

In addition, the legal gambling sector channels money into initiatives designed to help excessive gamblers (both legal and illegal) and society in general such as the Integrated Centre on Addiction Prevention and Treatment (ICAPT), contributions to the Ping Wo fund (dealing with excessive gamblers) and general contributions to taxation and charity. The HKJC contributes some three quarters of its revenue to tax and charitable causes.

In contrast, the illegal gambling market offers few such safeguards and makes no (known) direct contribution to initiatives aimed at addressing gambling addiction or general taxation.

While evidence of behaviours in illegal gambling establishments is limited, past international work has pointed to the fact that illegal operators play upon human frailties, encouraging feelings of power and omnipotence amongst winners and presenting them with gifts (e.g. the best food and cigarettes) in an effort to encourage further gambling (Bensimon et al. 2013).

In Hong Kong, Chinese male gamblers are likewise known to enjoy acts of bravado, bringing followers on trips to Macau and distributing large winnings in order to demonstrate their prowess (Chan and Ohtsuka 2011). The fact that illegal operators play upon such vulnerabilities, rather than seeking to ameliorate them, may increase the risk of gambling addiction amongst illegal gamblers.

Accordingly, expanding the range of legal gambling options may help reduce exposure to illegal gambling operators, the harmful practices which they could encourage among patrons and the attendant social ills. Given the high proportion of excessive gamblers within the ranks of illegal gamblers, bringing such gamblers ‘back into the fold’ may help break the cycle of gambling addiction and its attendant social effects.

A second perspective is the financial one. As a part of the abovementioned survey work, both legal and illegal gamblers were asked a series of questions about how their gambling spend would change in the event of a series of (independent) regulatory changes on gambling. This allowed for modelling of the impacts of these regulatory changes by 2025–2026, including both changes in legal gambling spending and the diversion of spending from illegal gambling markets, as illegal gamblers are attracted to the expanded range of legal betting options.

Full details of the modelling approach are provided in Oxford Economics (2016). The modelling represents only an initial estimate based on the survey results and further confirmatory research is required. Further, no judgement is made on the desirability of the modelled options.

Nonetheless, this work found that allowing Hong Kong gamblers to have more football betting options was the single largest revenue generator of the options studied, providing HK$6.1 billion by 2025–2026 of which HK$5.3 million were estimated to be displaced from the illegal market. Likewise, offering betting products on Hong Kong football league matches was found to generate HK$4.8 million in revenue, with HK$4.1 million of this coming from the illegal market. Conversely, construction of a Hong Kong casino was found to displace little illegal gambling revenue. This is probably due to the fact that the illegal casino market is itself small—because such gamblers already have a legal outlet through casino gambling in Macau.

These options are also associated with additional tax revenues. Tax is not an economic benefit, but a transfer between members of society. However, the proposition that such funds are better distributed back into Hong Kong society through taxes, rather than into the hands of illegal gambling operators is one that few would disagree with.

These considerations lead to the question of the economic effects of illegal gambling. Empirical studies on the long term economic effects of crime and the application of criminal proceeds rival those of illegal gambling in terms of their scarcity. While it is difficult to be certain in the case of Hong Kong, past work has suggested that criminal investment patterns tend to be sub-optimal with a particularly large over-emphasis on the real estate market (Kruisbergen et al. 2015; Unger et al. 2006; Walker 2007). While some funds may be invested back into the local housing market, given the need to avoid detection and strong mainland links, it is possible that Hong Kong illegal gambling operators send substantial amounts of money to offshore real estate. If so, this sub-optimal transfer of wealth could act to slow Hong Kong’s longer term growth via the inefficient allocation of investment.

## Discussion

Empirical work on illegal gambling is rare. This study is intended as a first step rather than as a last word. Much more needs to be done in undertaking research on the characteristics of illegal gamblers and the policy implications of such activity. However, in order to frame this initial analysis, we set out to test several hypotheses on the nature of illegal gamblers in Hong Kong.

In terms of *H*_*1*_, we find evidence that illegal gambling tends to attract individuals with a particularly avid interest in gambling. Hong Kong illegal gamblers bet more frequently and bet larger amounts than their legal counterparts. They are more attracted by good odds and betting flexibility than legal gamblers. Indeed the vast majority of ‘illegal’ gamblers are also keen participants in the legal market. This is consistent with the suggestions of de Bruin et al. (2005) and Binde (2011) about the characteristics of the illegal market. In particular, it may be that, as suggested by Binde, the legal market simply is not sufficient to cater for the diverse tastes of illegal gamblers.

We also find support for H_2_. Consistent with the international work of de Bruin et al. (2005), Binde (2011), and Volberg (2001), excessive gamblers are indeed overrepresented within the ranks of illegal gamblers in Hong Kong, with 56% of illegal gamblers being identified as excessive ones.

The causal link between excessive gambling and illegal gambling remains unclear. Since many excessive gamblers don’t engage in illegal gambling, one possibility is that a subset of excessive gamblers find their way into illegal gambling due to their avid interest in gambling. This is consistent with the suggestion of authors such as Binde (2011). However further research will need to be done to establish this more clearly.

The evidence for H_3_ is more nuanced. A greater proportion of illegal gamblers appear to have suffered from at least one of the identified social problems than is the case for legal gamblers. However, the relevant sample sizes are relatively small, and results are less clear for individual issues. This is not surprising in itself. The difficulty of finding an adequate sample to investigate the social problems due to gambling even in international work with very large initial sample sizes has been noted above. Reticence to self-identify and the issue of causality add additional layers of complexity (though it should be noted that the relevant survey questions explicitly asked illegal gamblers if these conditions were due to their gambling).

Nonetheless, noting these caveats, the survey results provide some tentative evidence of a greater potential susceptibility of illegal gamblers to social problems which might be expected due to the relatively high proportion of excessive gamblers in their ranks. In particular, susceptibility to conditions such as relationship breakdown, emotional stress of partners or children and debt accumulation are consistent with past literature (Productivity Commission 1999; Kunzi et al. 2004; Yip et al. 2007).

Further research would be necessary to expand on these tentative results and on the findings of this exploratory survey in general. One approach to undertaking such work would be a fully random sample of the population which would pick up illegal gamblers. However, a random sample would require a very large sampling ‘base’ to get at the sub-population of interest (illegal gamblers) which, in turn, requires a commitment to incur a major amount of time and resources to obtain information on only a small share of the overall gambling population.

As an alternative, a population survey of middle aged males—the group known to be the most likely to gamble illegally—could be conducted. While this might also require a relatively large sample size, such an approach would be more efficient and practical than a purely random approach and allow for a further exploration of the study themes discussed above.

Both past research and the current study could be also used to help inform future policy decisions. The lack of constraints in illegal gambling environments, as alluded to in the work of Bensimon et al. (2013), could spur on excessive gamblers and amplify their problems. This is in contrast to the direct and indirect ways in which legal gambling establishments attempt to address the issue of excessive gambling.

Previous studies have also alluded to the social harms associated with excessive gambling and both the current study and past work have indicted the overrepresentation of excessive gambles within the ranks of illegal gamblers. Liberalisation may offer one approach to ameliorating some of these issues. Expanding the range of legal gambling options would draw illegal gamblers away from the damaging influence of illegal operators into the legal word, where controls help restrain excessive and damaging gambling behaviour. Moreover, while more work needs to be done to confirm these initial results, some liberalisation measures in areas such as football betting could provide financial benefits and keep money out of the hands of illegal operators to be redistributed back to society through taxation.

## Conclusion

This study provides some initial insights into the nature of illegal gambling in Hong Kong, including its social and financial implications. Understanding the nature of illegal gambling is a first step in determining how to tackle the problem. Further research is required to confirm and extend its results, which will aid in helping to make important policy decisions regarding the regulation of Hong Kong gambling into the future.

## Additional file

**Additional file 1.** Survey questionnaire.

## Footnotes

1 Previous gambling studies in Hong Kong and elsewhere have used a variety of different gambling screens such as DSM-4 which differ from each other in their classification of ‘problem gamblers’. In order to aid comparability with past work, the term ‘excessive gamblers’ used in this study refers to a combination of the ‘moderate risk’ and ‘problem gamblers’ classifications under the Problem Gambling Severity Index (PGSI). (In turn, ‘Moderate risk’ gamblers under the PGSI equate to ‘problem gamblers’ in earlier Hong Kong, Macau and Singapore studies which used DSM-4 as a screen. ‘Problem gamblers’ under PGSI equates to pathological gamblers classified in these studies using DSM-4.) Low risk gamblers under the PGSI have been termed ‘low level excessive gamblers’ for this study. While other studies often refer to terms such as ‘problem gambling’, to avoid confusion the term ‘excessive gambling’ has also been used consistently when referring to the results of these studies. An exception is made when a past study clearly differentiates between sub-sets such as pathological gamblers.

2 Studies such as Wong et al. (2014) do report on issues such as suicidal intentions of Hong Kong illegal gamblers but they are simply one category of study rather than the focus of this work. The Hong Kong Polytechnic University’s 2016 survey of gambling in Hong Kong, Hong Kong Polytechnic University (2017), reports that 0.5% of the population gambles illegally, though 0.8% report engaging in “credit betting”. While the characteristics of illegal gamblers are examined in that report, the number of identified illegal gamblers equated to 6 out of 1258 respondents, limiting what conclusions could be drawn. As indicated in that survey, one difficulty is reticence to truthfully report on such gambling activities, which could contribute to underreporting. Broad population surveys are often hampered by such problems and the difficulties of adequately sampling such sub-groups: a parallel issue arises with attempts to study excessive gambling through such surveys. The survey we undertook for this paper asked respondents which gambling channels they had used (whether through the HKJC or otherwise) and avoided directly questioning whether they had participated in illegal gambling.

3 This included a major national survey of gambling habits in the Australian general population involving 1225 regular gamblers, 1290 non-regular gamblers and 983 non-gamblers. In addition, a separate survey involved 404 clients in counselling centres. Both surveys included various questions asking respondents if a variety of social, financial and relationship issues were caused by their gambling. This was supplemented by expert testimony on the links between a range of economic social and financial issues and excessive gambling.

4 Results differed across the range of psychiatric conditions and overall, 55.8% of respondents reported the condition existed before the onset of pathological gambling. However note that this result relates to pathological gamblers rather than the broader group of excessive gamblers.

5 For the purposes of this study, an illegal gambler is defined as someone within Hong Kong who places a bet with an operator other than the HKJC. The HKJC is the only authorised gambling operator licensed by the Hong Kong government (Hong Kong Jockey Club website “Illegal Gambling in Hong Kong” accessed 20 August 2017 http://www.hkjc.com/responsible-gambling/en/anti-illegal/index.aspx). Illegal gambling channels may include unlicensed operators in Hong Kong, offshore operators outside of Hong Kong (excluding Macau casinos) and online operators both inside and outside Hong Kong. More specifically, the survey classified ‘gamblers’ as those who admitted to gambling of any sort within the previous 12 months, while ‘illegal gamblers’ were those who admitted to gambling within Hong Kong within the past 12 months through channels not licenced by the SAR government.

6 95 of the 103 illegal gamblers were in fact “dual gamblers”, participating in both legal and illegal gambling. This is consistent with the international literature that finds illegal gamblers straddle both the legal and illegal modes of gambling. Nonetheless, they will be referred to as “illegal” gamblers throughout the rest of this paper for purposes of clarity and to distinguish them from purely legal gamblers.

7 While the survey provider noted that the use of convenience sampling may introduce some sampling bias in respect of illegal gamblers, they also noted that it is uncertain how material this is, given that a study of the underlying population of illegal gamblers does not exist. Further research would assist in clarifying this issue. As discussed below, however, it is notable that the profile of gamblers developed through this study is consistent with that established by international work (e.g. relatively high proportion of excessive gamblers, high representation of middle aged males).

8 A Mann–Whitney U test was used in respect of differences in gambling spend.

9 Two versions of the legal gambler results were developed: a population-weighted version, using past gambling population data and an unweighted version. Since there was little prior indication of the actual population distribution of illegal gamblers, the unweighted legal survey results were compared to the unweighted illegal gambler results. Discussions with the survey provider also indicated that weighting did not make a major difference to the legal gambler results.

10 Indeed, the difficulties of recruiting illegal gamblers are well illustrated by de Bruin et al. (2005). Undertaking a major random survey, the authors contacted 20,000 addresses, from whom 5575 respondents were recruited for their survey (a response rate of 28%). (Of these, 179 declared they were former illegal gamblers, with 34 declaring they were current illegal gamblers.) In addition, the authors estimated that some 70,000 people in the Netherlands suffered from gambling addiction, although this equated to only 28 participants in the main survey—below the usual benchmark for sample size (n = 30). They therefore took an approach which mirrored the current study and undertook supplementary face-to-face interviews of 413 gamblers, recruited from the main survey, various gambling venues, advertisements or other means. This yielded 42 excessive gamblers and 39 past excessive gamblers. In addition, some 10% of the face to face sample (42 gamblers) were current illegal gamblers, while 17% (71 were ex-illegal players).

11 Unless otherwise indicated, references to statistical significance for the work underlying this paper indicate that the difference was significant at the 5% level using a Chi Square test. Unless otherwise indicated, the sample sizes for illegal gamblers used in the comparisons for the work relating to this paper was 103 and that of legal gamblers 497.

12 Based on a Mann–Whitney U test at the 5% significance level.

13 Based on a Chi-Square Test at the 5% significance level.
